# ﻿*Hedyotislongiramulis* (Rubiaceae), a new species from south China

**DOI:** 10.3897/phytokeys.230.87675

**Published:** 2023-08-18

**Authors:** Yi-Da Xu, Ying Zhang, Rui-Jiang Wang

**Affiliations:** 1 State Key Laboratory of Plant Diversity and Specialty Crops, South China Botanical Garden, Chinese Academy of Sciences, Guangzhou, Guangdong 510650, China South China Botanical Garden, Chinese Academy of Sciences Guangzhou China; 2 College of Life Sciences, University of Chinese Academy of Sciences, Beijing 100049, China University of Chinese Academy of Sciences Beijing China; 3 Center of Conservation Biology, Core Botanical Gardens, Chinese Academy of Sciences, Guangzhou 510650, China Core Botanical Gardens, Chinese Academy of Sciences Guangzhou China

**Keywords:** Chloroplast genome, *Hedyotis*-*Oldenlandia* complex, phylogeny, taxonomy

## Abstract

*Hedyotislongiramulis***sp. nov.** (Rubiaceae) is described from Guangdong Province, China. It is similar to *H.caudatifolia* but differs in having puberulent, more or less tetragonal and decussately sulcate juvenile stems, waxy leaf surface, short inflorescence peduncles, high length ratio of corolla lobe to tube, and subglobose capsules. The phylogenetic analysis reveals that *H.longiramulis* is sister to *H.pubirachis*. Dimorphism concerning pollen size was observed in the heterostylous flowers. The complete chloroplast genome of the new species comprises a typical quadripartite structure of 153,616 bp in length, with two inverted repeats of 25,457 bp, a large single-copy of 85,050 bp and a small single-copy of 17,652 bp. It contains 112 unique genes, including 79 protein-coding genes, 29 tRNA genes, and four rRNA genes, the GC content of the chloroplast genome is 32.4%. The new species is provisionally evaluated as “Least Concern” because it is common and well-protected in two Provincial Nature Reserves.

## ﻿Introduction

The genera *Hedyotis* L. and *Oldenlandia* L. are two taxonomically disputed genera and usually considered as a taxonomic complex in the tribe Spermacoceae of Rubiaceae. These two genera include more than 500 species distributed in tropical and subtropical regions worldwide (Dutta and Deb 2004). Taxonomical treatment of several genera within the tribe, especially regarding to generic delimitation, has much been debated (e.g., Lamarck 1792; Willdenow 1797; Bremekamp 1952; Dutta and Deb 2004). Recent phylogenetic analyses proved that the *Hedyotis*-*Oldenlandia* complex was polyphyletic and a narrow generic delimitation was then proposed accordingly (Guo et al. 2013; Gibbons 2020). Currently, *Hedyotis* s. str. is characterized by having an erect and robust herbaceous or shrubby habit, homo- or heterostylous flowers, triangular or ovate stipules with serrate marginal glands and tipped colleters, mostly diplophragmous capsules (loculicidal dehiscence first and then septicidal dehiscence along the septum) and fruticosa-type seeds (dorsiventrally flattened, lenticular with irregularly narrow wing-like margin). The distribution center of *Hedyotis* s. str. is the Asian-Pacific region (Terrell and Robinson 2003).

With the rapid development of high-throughput sequencing technologies, whole chloroplast genome dataset is increasingly used for simulating phylogenetic relationships (Liu et al. 2018; Song et al. 2019; Charr et al. 2020; Rono et al. 2020; Zhang et al. 2021). However, all of the present molecular phylogenetic analyses on the *Hedyotis*-*Oldenlandia* complex are based on a handful of nuclear or chloroplast DNA markers. Therefore, a more reliable phylogenetic relationship with robust support based on the whole chloroplast genome dataset is strongly anticipated. But unfortunately, for *Hedyotis* s. str., only the whole chloroplast genome dataset of *H.ovata* Thunb. ex Maxim. is available (MK203877) up to now (Zhang et al. 2019).

During a field collection in Guangdong Ehuagnzhang Provincial Nature Reserve, we found a sub-shrubby species of *Hedyotis* s. str. with purplish and puberulent young stems and long axillary branches. It is similar to *H.caudatifolia* Merr. & F.P.Metcalf with respect to its erect subshrubby habit, ovate to lanceolate leaf shape, and long lateral branches bearing several terminal and axillary inflorescences, but conspicuously differs by its puberulent, more or less tetragonal and decussately sulcate juvenile stems. After detailed morphological comparison and phylogenetic analysis, we confirm that this species is a hitherto undescribed one.

## ﻿Materials and methods

### ﻿Morphological examination

Morphological data of the new species was observed on living individuals and herbarium specimens deposited at IBSC and CANT (herbarium code follows https://sweetgum.nybg.org/science/ih/).

For micromorphology, scanning electron microscopy (SEM, JSM-6360LV) was applied under 15.00 kV accelerating voltage. Pollen grains were put in 70% alcohol, washed by an ultrasonic cleaner (WIGGENS UA10MFD, 100W, 59KHZ) for 5 min, and then centrifuged at 8000 rpm for 5 min. After this, we removed the supernatant and added 70% alcohol to the sediment. These steps were repeated three times. Finally, the pollen suspension was dropped on the sample stubs with conductive double sided adhesive carbon tapes. The pollen samples were gilded by sputter coater (LEICA EM ACE600, 10 μm, 20 mA) once dried in room conditions. Seed samples were cleaned using the same method as for pollen grains and then transferred to sample stubs for gilding after drying. Leaf material was cleaned by brushing lightly and rinsing gently in warm water and then transferred to sample stubs after drying.

Pollen terminology for description followed Hesse et al. (2009), seed terminology followed Neupane et al. (2015), and foliar epidermal terminology followed David (1974).

### ﻿Conservation assessment

The conservation assessment was undertaken according to the guidelines for assessing the conservation status of species (IUCN 2022). Estimation of the extent of occurrence (EOO) and area of occupancy (AOO) were performed in GeoCAT (Bachman et al. 2011) with 2 × 2 km grid cells.

### ﻿Genomic DNA extraction and sequencing

Leaf material for DNA extraction was dried in silica gel. Total DNA was extracted using the modified cetyltrimethylammonium bromide (CTAB) protocol (Doyle 1991). Primers for polymerase chain reaction (PCR) are listed in Table 1, and the methods for PCR followed Guo et al. (2011). PCR products were purified and sequenced by Sangon Biotech Limited Company (Shanghai, China). For whole genome sequencing, the DNA samples were sent to Beijing Genomics Institute (Shenzhen, China) for genomic library construction and *de novo* sequencing (paired-end, PE=150 bp) using the BGISEQ-500. Raw reads were filtered and trimmed using SOAPnuke v.1.5.6 with software parameters “-n 0.01 -l 20 -q 0.3 -A 0.25 --cutAdaptor -Q 2 -G --polyX 50 --minLen 150”.

**Table 1.** Primers used for PCR in the present study.

DNA region	Primer name	Sequence	References
ITS	P17	5’-CTACCGATTGAATGGTCCGGTGAA-3’	Popp and Oxelman 2001
	26S-82R	5’-TCCCGGTTCGCTCGCCGTTACTA-3’	
*petB*-*petD*	PIpetB1365F	5’-TTGACYCGTTTTTATAGTTTAC-3’	Löhne and Borsch 2005
	PIpetD738R	5’-AATTTAGCYCTTAATACAGG-3’	
*rps16*	rps16F	5’-GTGGTAGAAAGCAACGTGCGACTT-3’	Oxelman et al. 1997
	rps16R3	5’-CGATAGACGGCTCATTGGGATA-3’	
*trnH-psbA*	trnH-05	5’-CGCGCATGGTGGATTCACAATCC-3’	Tate and Simpson 2003
	psbA3	5’-GTTATGCATGAACGTAATGCTC-3’	Sang et al. 1997
*trnL-F*	TabC	5’-CGAAATCGGTAGACGCTACG-3’	Taberlet et al. 1991
	TabF	5’-ATTTGAACTGGTGACACGAG-3’	

### ﻿Chloroplast genome assembly and annotation

A total of 2 Gb clean reads were obtained and assembled using GetOrganelle v.1.7.3.5 (Jin et al. 2020). With reference to *H.ovata* (GenBank: MK203877), the genome was first annotated using GeSeq (https://chlorobox.mpimp-golm.mpg.de/geseq.html) (Tillich et al. 2017) and PGA (Qu et al. 2019), and then manually adjusted using Geneious v.11.0.3. A circular map of the chloroplast genome was drawn using OGDRAW v.1.3.1 (https://chlorobox.mpimp-golm.mpg.de/OGDraw.html) (Greiner et al. 2019).

### ﻿Molecular phylogenetic analyses

Twenty-three morphologically similar and sympatric *Hedyotis* taxa, as well as two accessions of the new species (see Table 2), were selected as ingroup operational taxonomic units (OTUs) for molecular phylogenetic analyses. Two Spermacoceae species, *Dentellarepens* (L.) J.R.Forst. & G.Forst. from Australia and *Pentodonpentandrus* Vatke from Zambia were chosen as outgroup OUTs (see Table 2).

**Table 2.** Taxa, vouchers, localities, and GenBank accession numbers of ITS, *petD*, *rps16*, *trnH-psbA* and *trnL-F* sequences for phylogenetic analysis.

Taxon	Voucher (herbarium)	ITS	*petD*	*rps16*	*trnH-psbA*	*trnL-F*
*Dentellarepens* J.R.Forst. & G.Forst	Australia: Andersson 2262 (GB)	AM939440	EU557693	AF333370	/	EU543091
*Hedyotisacutangula* Champ. ex Benth.	China: unknown BW21 (CUHK)	HQ148749	/	HM752907	HM640307	HM752822
*Hedyotisacutangula* Champ. ex Benth.	China: Ruijiang Wang HA-02 (IBSC)	JX111197	JX111085	JX111241	JX111160	JX111316
*Hedyotiscantoniensis* F.C.How ex W.C.Ko	China: Ruijiang Wang et al. 1250 (IBSC)	JF699912	JF700061	JX111247	JF699773	JX111322
*Hedyotiscaudatifolia* Merr. & F.P.Metcalf	China: Ruijiang Wang et al. 1229 (IBSC)	JF699915	JF700064	JX111255	JF699776	JX111328
*Hedyotiscaudatifolia* Merr. & F.P.Metcalf	China: Ruijiang Wang et al. 1269 (IBSC)	JF699916	JF700065	JX111256	JF699777	JX111329
*Hedyotiscommunis* W.C.Ko	China: Bo Li LB0172 (IBSC)	JX111208	JX111094	JX111257	JX111167	JX111330
*Hedyotisconsanguinea* Hance	China: Ruijiang Wang 1254 (IBSC)	JF699923	JF700071	JX111258	JF699783	JX111331
*Hedyotiseffusa* Hance	China: Ruijiang Wang et al. 1268_1 (IBSC)	JF699933	JF700083	JX111262	JF699790	JX111335
*Hedyotisexserta* Merr.	China: Guobin Jiang and Xinxin Zhou 1124 (IBSC)	MT345066	MT347606	MT792387	MT792403	MZ514116
*Hedyotisinterrupta* G.B.Jiang & R.J.Wang	China: Guobin Jiang and Xinxin Zhou 1136_2 (IBSC)	MT345072	MT347612	MT792393	MT792409	MZ514117
*Hedyotisloganioides* Benth.	China: Ruijiang Wang 1253-1 (IBSC)	JF699910	JF700059	JX111246	JF699771	JX111320
*Hedyotislongiexserta* Merr. & F.P.Metcalf	China: Mingdeng Yuan et al. YS60 (IBSC)	MW396581	MW405435	MW405424	/	MZ514123
*Hedyotislongipetala* Merr.	China: Ruijiang Wang 1334 (IBSC)	JX111216	JX111102	JX111268	JX111175	JX111342
*Hedyotislongiramulis* Y.D.Xu & R.J.Wang	China: Yida Xu and Fan Su AP0138 (IBSC)	MZ326005*	MZ425928**	MZ425928**	MZ425928**	MZ425928**
*Hedyotislongiramulis* Y.D.Xu & R.J.Wang	China: Dan Liang et al. WP1366 (IBSC)	MZ411390*	MZ403800*	MZ417507*	MZ403809*	MZ417501*
*Hedyotismatthewii* Dunn	China: Ruijiang Wang et al. 1251 (IBSC)	JF699900	JF700049	JX111243	JF699761	JX111318
*Hedyotisnankunshanensis* R.J.Wang & S.J.Deng	China: Ruijiang Wang et al. 1688 (IBSC)	JN975969	JN975964	OQ723460*	OQ723461*	OQ723462*
*Hedyotisnanlingensis* R.J.Wang	China: Mingdeng Yuan et al. YS228 (IBSC)	MW396579	MW405437	MW405426	MZ514110	MZ514124
*Hedyotisovata* Thunb. ex Maxim.	China: Guobin Jiang et al. 1508 (IBSC)	MZ326003	MZ403799	MZ343053	MZ403807	MZ403793
*Hedyotispuberulifolia* Y.D.Xu & R.J.Wang	China: Ruijiang Wang and Yida Xu 6216 (IBSC)	MW169047	MW196744	OQ723463*	OQ723464*	OQ723465*
*Hedyotispubirachis* Y.D.Xu & R.J.Wang	China: Yida Xu and Fan Su AP0147 (IBSC)	MW264177	MW266052	MZ447121	MZ447124	MZ447126
*Hedyotispulcherrima* Dunn	China: Ruijiang Wang 1233-1 (IBSC)	JF699946	JF700096	JX111274	JF699801	JX111348
*Hedyotistaishanensis* G.T.Wang & R.J.Wang	China: Yida Xu et al. WP1330 (IBSC)	MZ479676	MZ514102	MZ514103	MZ514108	MZ514121
*Hedyotistenuipes* Hemsl.	China: Ruijiang Wang 1234_1 (IBSC)	JF699960	JF700110	JX111280	JF699812	JX111354
*Hedyotisxanthochroa* Hance	China: Ruijiang Wang 1361 (IBSC)	JX111227	JX111110	JX111286	JX111183	JX111361
*Hedyotisxinyiensis* X.Guo & R.J.Wang	China: Ruijiang Wang 1182 (IBSC)	JF699970	JF700120	JX111288	JF699820	JX111362
*Hedyotisyangchunensis* W.C.Ko & Zhang	China: Ruijiang Wang 1270-1 (IBSC)	JF699972	JF700122	JX111290	JF699821	JX111364
*Pentodonpentandrus* Vatke	Zambia: Dessein et al. 598 (BR)	AM939528	EU557759	EU543066	/	EU543154

*indicates that the sequences are newly obtained by PCR sequencing. **indicates that the sequences are newly obtained by whole genome sequencing.

Five DNA markers (ITS, *petD*, *rps16*, *trnH-psbA* and *trnL-F*) were employed to reconstruct the phylogenetic trees. Sequences were aligned using MAFFT v.7.017 (Katoh et al. 2002) and then concatenated together in Geneious. Maximum Likelihood (ML) analyses were accomplished with IQ-TREE v.2.0 (Nguyen et al. 2015). The best-fit nucleotide substitution model of GTR+F+R2 was selected by using ModelFinder (Kalyaanamoorthy et al. 2017). Bayesian inference (BI) analyses were accomplished with MrBayes v.3.1.2 (Ronquist et al. 2012). GTR+G+I was selected to be the best-fit nucleotide substitution model by MrModeltest v.2.3 (Nylander 2004). The sampled species along with their voucher information and GenBank accession numbers are listed in Table 2.

## ﻿Results

### ﻿A new species based on morphological and molecular evidence

#### ﻿Morphology

During our examination of herbarium material, we found that *Hedyotislongiramulis* was often misidentified as either *H.caudatifolia* or *H.communis* W.C.Ko because of the subshrubby habit, the ovate to lanceolate leaves and the triangular stipules. A detailed morphological comparison is therefore provided to elucidate the differences among them (Table 3).

**Table 3.** Diagnostic characters of *Hedyotislongiramulis*, *H.pubirachis* (sister species in molecular analysis), *H.caudatifolia* and *H.communis* (two morphologically similar species).

Characters	*H.longiramulis*	*H.caudatifolia*	*H.communis*	*H.pubirachis*
Stem	more or less tetragonal and decussately sulcate at juvenile internodes, puberulent	terete or slightly flattened, glabrous	terete or slightly flattened, glabrous	terete with inconspicuous ridges, glabrous
Leave surface	waxy on both side	glabrous on both side	glabrous on both side	glabrous on both side
Petiole length (mm)	5–15 on main stem and 2–5 on lateral branches	3–15	subsessile	3–10
Stipules	triangular, densely puberulent abaxially	triangular, glabrous abaxially	narrowly triangular, glabrous abaxially	triangular to broadly ovate, glabrous abaxially
Inflorescences	growing on lateral branches, terminal and axillary in the upper nodes	growing on lateral branches, terminal and axillary in the upper nodes	growing on main stem and on lateral branches, strictly axillary	growing on main stem and on lateral branches, terminal and axillary in the upper nodes
Peduncle length (cm)	0.5–2.0	2.0–10.0	0.5–2.5	2.5–7.0
Calyx lobes length (mm)	ca. 0.9	0.8–1.0	2–3	ca. 0.5
Calyx lobes shape	ovate-triangular with blunt or rounded apex	triangular with acute apex	narrowly triangular with acute apex	broadly triangular
Ratio of calyx lobe length to its basal width	ca. 1:1	1–1.5:1	2.5–3:1	ca. 0.8:1
Corolla tube length (mm)	3.5–3.8	3.0–4.0	4.0–5.0	2.8–3.3
Corolla lobe length (mm)	3.5–3.8	2.0–2.7	2.5–4.0	2.0–2.2
Length ratio of corolla lobe to tube	0.9–1.0	ca. 0.8	0.6–0.8	ca. 0.7
Capsule shape	subglobose	ellipsoid-oblong or ellipsoid	obovoid or subglobose	ellipsoid to subglobose

#### ﻿Molecular analysis

**Figure 1.**
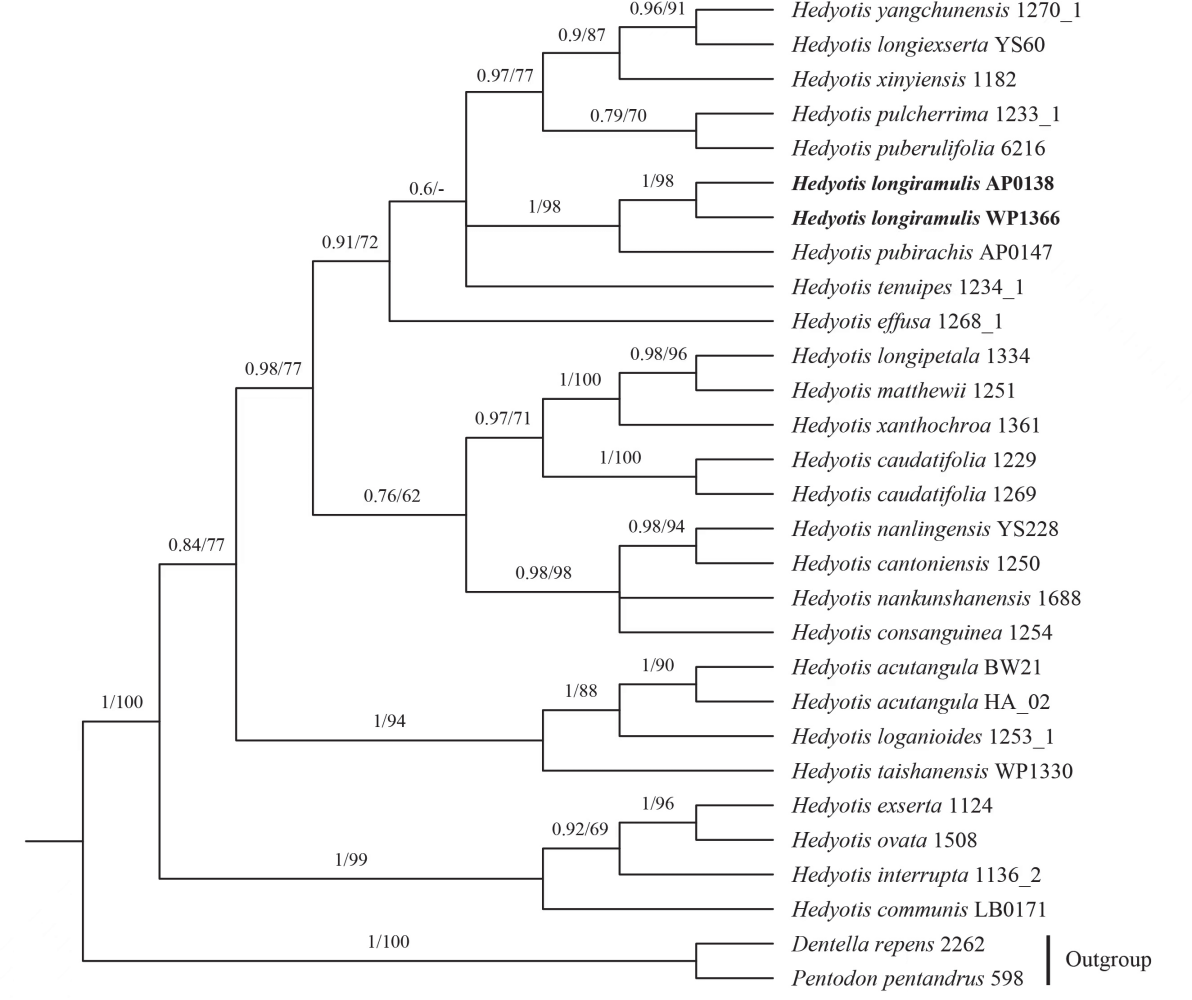
Phylogenetic relationships of *Hedyotis* based on combined nuclear ITS and four plastid markers (*petD*, *rps16*, *trnH*-*psbA* and *trnL*-*F*). Bootstrap values (BS≥50%, right) and Bayesian Posterior Probabilities (PP≥0.5, left) are labeled above the branches. Field collection numbers are labeled after species names.

BI and ML analyses based on the combined nuclear ITS and four plastid markers (*petD*, *rps16*, *trn*H-*psbA* and *trnL*-*F*) result in the same tree topology. The two accessions of the new species form a monophyletic clade that is sister to *H.pubirachis* Y.D.Xu & R.J.Wang with robust support (PP = 1, BS = 98) (Fig. 1). The two species share common characters, such as subshrubby habit and ovate to lanceolate leaf shape, but differ in other characters. A comparison of the morphological characters is given in Table 3.

### ﻿Taxonomic treatment

#### ﻿ Hedyotis longiramulis

Taxon classificationPlantaeGentianalesRubiaceae

Y.D.Xu & R.J.Wang sp. nov.

8545C705-65AD-5BE6-AB0C-91E0A73FC442

urn:lsid:ipni.org:names:77325483-1

**Figure 2.**
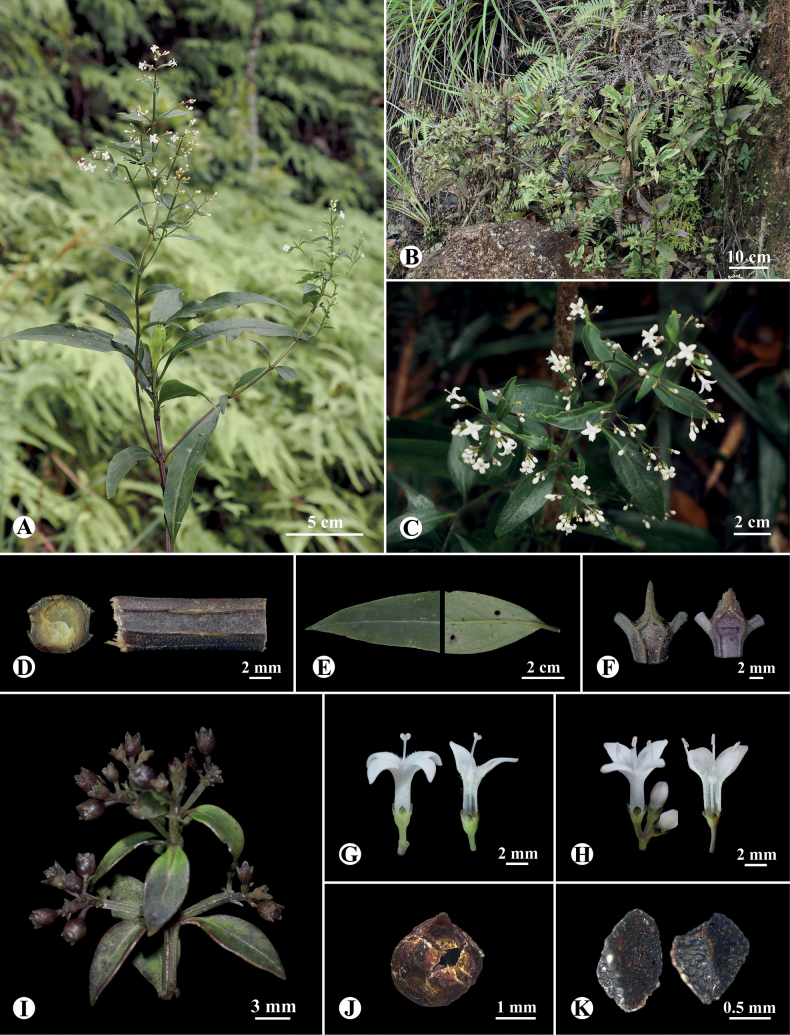
*Hedyotislongiramulis* Y.D. Xu & R.J. Wang **A** habit **B** habitat **C** inflorescences **D** part of stem (right) and its transverse section **E** adaxial (left) and abaxial (right) surgaces of leaf **F** Stipules **G** long-styled flower (left) and its longitudinal section (right) **H** short-styled flower (left) and its longitudinal section (right) **I** infructescence **J** diplophragmous capsule **K** dorsal (left) and ventral (right) view of seeds.

**Figure 3.**
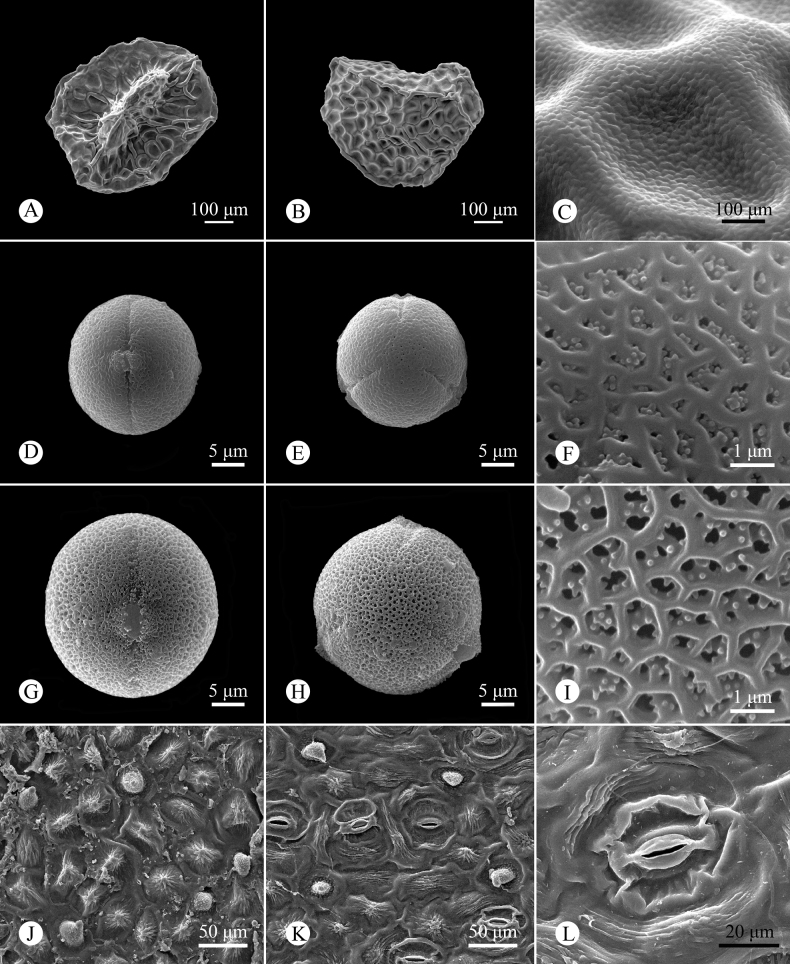
Micromorphology of seed, pollen and leaf epidermis of *Hedyotislongiramulis* using SEM**A–C** ventral view, dorsal view, and surface ornamentation of seeds, respectively **D, G, E, H, F, I** equatorial view, polar view, and reticulate ornamentation of pollen grains, respectively **J–L** leaf epidermis, adaxial and abaxial surfaces, and stomatal apparatus, respectively **A–C, J–L***Yi-Da Xu & Fan Su AP0138***D–F***Rui-Jiang Wang & Yi-Da Xu 6540*, long-styled flower **G–I***Rui-Jiang Wang & Yi-Da Xu 6541*, short-styled flower.

Figs 2 , 3 鹅凰嶂耳草 (é Huáng Zhàng ěr Căo)

##### Type.

China. Guangdong Province: Yangchun City, Bajia Town, Guangdong Ehuangzhang Provincial Nature Reserve, roadsides, 21°52'N, 111°25'E, elev. 643 m. April 9, 2021, *Y.D. Xu & R.J. Wang 6540* (holotype: IBSC [IBSC0865777!]; isotype: IBSC [IBSC0865778!]).

##### Diagnosis.

The species is similar to *H.caudatifolia* in having a subshrubby habit, ovate to lanceolate leaves, and long lateral branches with several terminal and axillary inflorescences, but differs from it by having puberulent, more or less tetragonal and decussately sulcate juvenile stems (versus glabrous and terete in *H.caudatifolia*), waxy leaf surface (versus non-waxy in *H.caudatifolia*), shorter peduncles (0.5–2.0 cm versus 2.0–10.0 cm in *H.caudatifolia*), a higher length ratio of corolla lobe to tube (0.9–1.0 versus approximately 0.8 in *H.caudatifolia*), and subglobose capsules (versus ellipsoid-oblong or ellipsoid in *H.caudatifolia*).

##### Description.

Perennial woody subshrubs, 40–120 cm tall. ***Stem*** more or less tetragonal and decussately sulcate at juvenile internodes, becoming terete with age, purplish, puberulent, branched at upper part. ***Leaves*** opposite, 5–16 × 1.5–4 cm on main stem and 1.0–6.5 × 0.3–1.5 cm on lateral branches, ovate to lanceolate, coriaceous, dark green adaxially, greyish-green or sometimes purplish abaxially, both surfaces waxy, apex acute or subacute, base cuneate or shortly decurrent; petiole 5–15 mm long on main stem and 2–5 mm long on lateral branches, waxy or puberulent; midrib depressed adaxially and prominent abaxially, secondary veins usually 5–6 on each side, sometimes indistinct adaxially; ***stipules*** 4–10 × 3–6 mm, triangular, apex acute to acuminate, margin sparsely glandular serrate, puberulent abaxially. ***Inflorescences*** growing on long lateral branches, terminal and axillary in the upper nodes, 1.5–3.5 cm long, cymose or paniculate-cymose; inflorescence axes tetragonal, sulcate; peduncles 0.5–2.0 cm long; bracts ca. 1 mm long, subulate. ***Flowers*** heterostylous, pedicels 0.9–2.0 mm long. ***Hypanthium*** ca. 1 mm long, obconic to subglobose; lobes 4, ca. 0.9 × 0.9 mm, ovate-triangular, blunt or rounded at apex. ***Corolla*** white or purplish, tube 3.5–3.8 mm long, glabrous abaxially and densely or sparsely pubescent adaxially; lobes 4, 3.5–3.8 × 1.8–2.2 mm, ovate-triangular; stamens 4, anthers ca. 0.9 mm long; stigma bilobed, ca. 0.5 mm long, subglobose, papillate. ***Long-styled flowers***: stamens included, filaments ca. 0.6 mm long, adnate to the middle part of corolla tube; style ca. 7.6 mm long, exserted, glabrous. ***Short-styled flowers***: stamens exserted, filaments ca. 2.8 mm long, adnate to the throat of corolla tube; style ca. 2.7 mm long, included, glabrous. ***Fruits*** capsular, ca. 2.0 mm in diameter, subglobose, glabrous, dehiscent diplophragmously; seeds several, ca. 1 mm long, cymbiform, with reticulate surface. (Fig. 3A–C.)

##### Distribution and habitat.

*Hedyotislongiramulis* is only known from Yangchun City of Guangdong Province, China. It grows mainly in damp places under broad-leaved forests, sometimes on roadsides at the elevation of 500–700 m. The associated species are mainly (Hance ex Benth.) Krass. (Melastomataceae), *Melastomasanguineum* Sims (Melastomataceae), *Dunniasinensis* Tutcher (Rubiaceae), *Dicranopterisampla* Ching & P.S.Chiu (Gleicheniaceae) and *Selaginelladoederleinii* Hieron. (Selaginellaceae).

##### Phenology.

Flowering from late March to July, fruiting from August to October.

##### Etymology.

The specific epithet “longiramulis” of the new species refers to its long lateral branches bearing many inflorescences.

##### Palynology.

The pollen grains of *Hedyotislongiramulis* are monads, isopolar, spheroidal, 3-colporate; the tectum is a double microreticulum, with a psilate suprareticulum and a microechinate infrareticulum. The pollen size is 22.5 (20.2–25.1) × 21.5 (19.0–22.8) μm with P/E value 1.04 in long-styled flowers (Fig. 3D–F) and 27.1 (25.2–29.3) × 27.1 (25.1–28.7) μm with P/E value 1.00 in short-styled flowers (Fig. 3G–I).

##### Foliar epidermal anatomy.

The epidermal cells on the upper (Fig. 3J) and lower (Fig. 3K) surface of leaves of *H.longiramulis* are irregularly polygonal, randomly arranged and have striated and papillate surface ornamentation, with the striations thickened at the middle of the periclinal walls, and the papillae conical, with granular ornamentation on the surface. The anticlinal walls are straight in epidermis cells of the upper leaf surface and undulate in those of the lower leaf surface.

The leaves of *H.longiramulis* are hypostomatic, with the stomata randomly orientated over most of the lower surface. The stomata are paracytic, ca. 56.5 (51.4–63.4) × 42.8 (37.1–52.1) μm in size (Fig. 3L).

##### Additional specimens examined

**(paratypes). China.** Guangdong Province: Yangchun City, Guigang Town, Baichong Provincial Nature Reserve, roadside, 13 Sept. 1990, Nian Liu et al. 424 (IBSC); ibid., 18 May 1991, Nian Liu et al. 1735 (IBSC). Yangchun City, Bajia Town, Guangdong Ehuangzhang Provincial Nature Reserve, mountain land and valley, 24 Oct. 1957, Kui Liang 69692 (CANT); ibid., 23 Oct. 1957, Bao-Han Liang 89654 (CANT); ibid., 11 Oct. 1990, Nian Liu et al. 866 and 899 (IBSC); ibid., 11 May 2001, Hua-Gu Ye et al. 5629 (IBSC); ibid., 7 Apr. 2019, Xin-Xin Zhou et al. ZXX0026 (IBSC); ibid., 12 Aug. 2020, Dan Liang et al. WP1366 (IBSC); ibid., 10 Sept. 2020, Yi-Da Xu & Fan Su AP0138 (IBSC); ibid., 9 Apr. 2021, Rui-Jiang Wang & Yi-Da Xu 6541 (IBSC).

##### Conservation status assessment.

So far 10 subpopulations of *Hedyotislongiramulis* were found in Yangchun City (AOO 40 km^2^, EOO 758 km^2^), Guangdong Province, and their habitats are well protected. About 60 mature individuals were found in each of these subpopulations (within 2 × 2 km grid cells). We therefore estimated that there are at least 600 mature individuals in this area. According to the criteria D1 of IUCN Red List Categories and Criteria (IUCN 2022), the species can be assessed as “Vulnerable”. However, many other subpopulations of this species may be found in similar habitat nearby the vouchers’ localities in the nature reserves. Considering that this species has no economic uses and that there are no plausible threats since it occurs in two protected reserves, we recommend to evaluate it as “Least Concern”.

### ﻿Characteristics of the chloroplast genome

**Figure 4.**
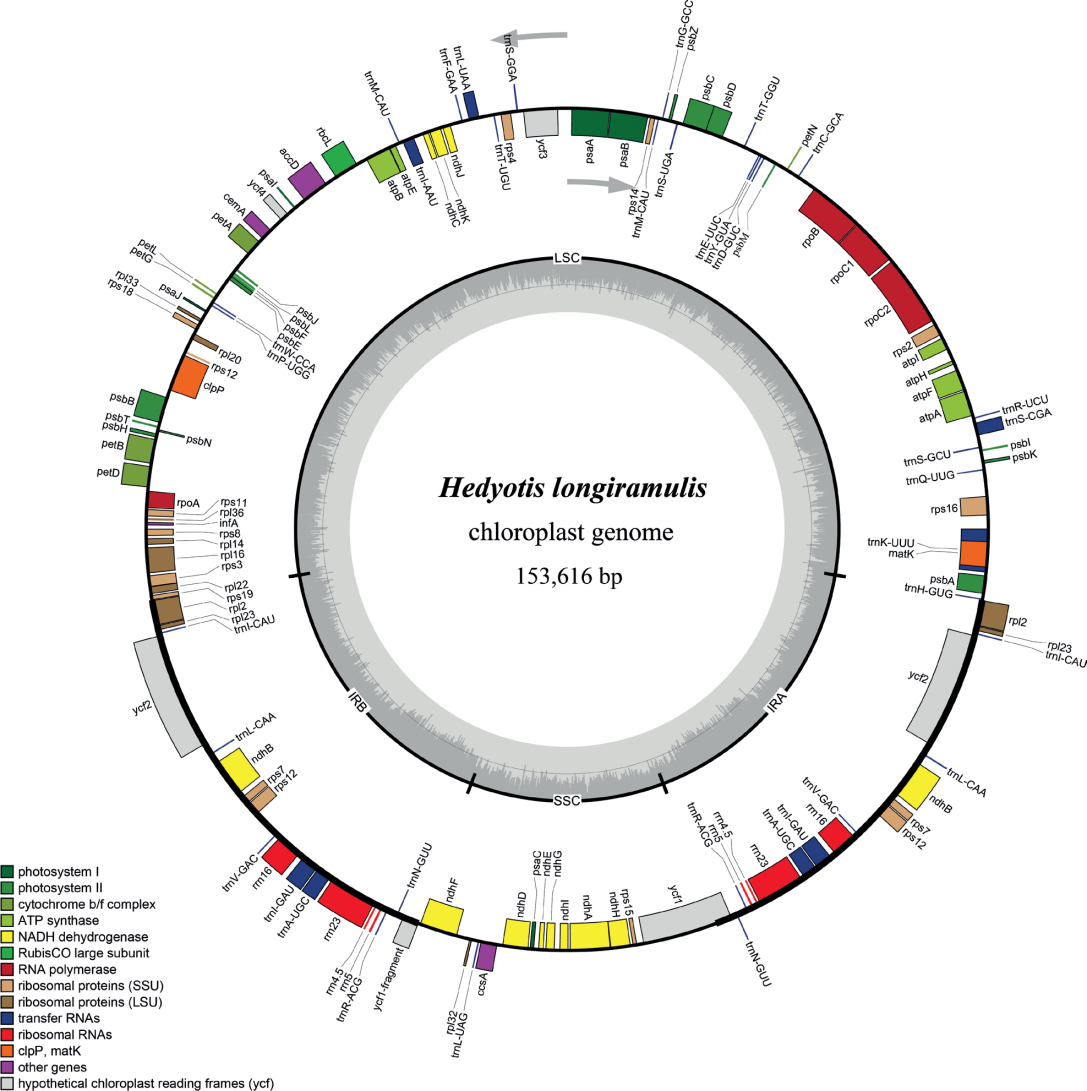
Chloroplast genome map of *Hedyotislongiramulis*. The thick lines on the outer complete circle identify the inverted repeat regions (IRa and IRb). The arrows indicate the transcription directions of the genes inside and outside of the circle. Genes belonging to different functional groups are color-coded. The dark gray in the innermost track corresponds to the GC content, the light gray to the AT content.

**Figure 5.**
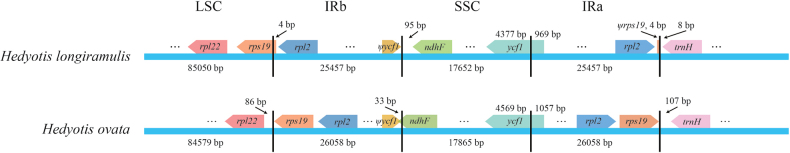
Sequence comparison of the IR/SC boundaries between *Hedyotislongiramulis* and *H.ovata*.

The size of the complete chloroplast genome of *H.longiramulis* is 153,616 bp (GenBank: MZ425928, Fig. 4) with a typical quadripartite structure, including a small single-copy region (SSC, 17652 bp), a large single-copy region (LSC, 85050 bp), and a pair of inverted repeat regions (IRs, 25457 bp). It contains 112 unique genes, and the GC content is 32.4% (Table 4). The *rps19*, *ycf1*, *ndhF*, *rpl2* and *trnH* genes were found nearby the IR/Single-Copy (SC) region boundaries. Compared with *H.ovata*, the IR of *H.longiramulis* contracted to include only 4 bp of the 5’ end of *rps19* (vs. entirely included and occurring twice in IRs of *H.ovata*), and excludes the entire *ndhF* and 100 bp of the intergenic region (vs. including 32 bp of the 3’ end of *ndhF* in *H.ovata*) (Fig. 5). Detailed characteristics and statistics of the chloroplast genomes are listed in Tables 4, 5.

**Table 4.** Characteristics of the chloroplast genomes of *Hedyotislongiramulis* and *H.ovata*.

	Characteristics	*H.longiramulis* GenBank: MZ425928	*H.ovata* GenBank: MK203877
Size (bp)	Total	153,616	154,560
	LSC	85,050	84,579
	SSC	17,652	17,865
	IR	25,457	26,058
Number of unique genes	Total	112	112
	Protein-coding genes	79	79
	rRNA genes	4	4
	tRNA genes	29	29
GC%	Total	32.4	32.6
	LSC	35.9	36.0
	SSC	32.4	32.6
	IR	43.5	43.4
	protein-coding sequences (CDS)	38.4	38.9

**Table 5.** Genes encoded in the chloroplast genome of *Hedyotislongiramulis*.

Category	Group of genes	Names of unique genes
Self-replication	tRNA genes	*trnA*-*UGC*, *trnC*-*GCA*, *trnD*-*GUC*, *trnE*-*UUC*, *trnF*-*GAA*, *trnfM*-*CAU*, *trnG*-*GCC*, *trnH*-*GUG*, *trnI*-*CAU*, *trnI*-*GAU*, *trnK*-*UUU*, *trnL*-*CAA*, *trnL*-*UAA*, *trnL*-*UAG*, *trnM*-*CAU*, *trnN*-*GUU*, *trnP*-*UGG*, *trnQ*-*UUG*, *trnR*-*ACG*, *trnR*-*UCU*, *trnS*-*GCU*, *trnS*-*GGA*, *trnS*-*UGA*, *trnT*-*GGU*, *trnT*-*UGU*, *trnV*-*GAC*, *trnV*-*UAC*, *trnW*-*CCA*, *trnY*-*GUA*
	rRNA genes	*rrn4.5*, *rrn5*, *rrn16*, *rrn23*
	Ribosomal small subunit	*rps2*, *rps3*, *rps4*, *rps7*, *rps8*, *rps11*, *rps12*, *rps14*, *rps15*, *rps16*, *rps18*, *rps19*
	Ribosomal large subunit	*rpl2*, *rpl14*, *rps16*, *rpl20*, *rpl22*, *rpl23*, *rpl32*, *rpl33*, *rpl36*
	DNA-dependent RNA polymerase	*rpoA*, *rpoB*, *rpoC1*, *rpoC2*
Photosynthesis	Photosystem I	*psaA*, *psaB*, *psaC*, *psaI*, *psaJ*, *ycf3*, *ycf4*
	Large subunit of rubisco	*rbcL*
	Photosystem II	*psbA*, *psbB*, *psbC*, *psbD*, *psbE*, *psbF*, *psbH*, *psbI*, *psbJ*, *psbK*, *psbL*, *psbM*, *psbN*, *psbT*, *psbZ*
	NADH dehydrogenase	*ndhA*, *ndhB*, *ndhC*, *ndhD*, *ndhE*, *ndhF*, *ndhG*, *ndhH*, *ndhI*, *ndhJ*, *ndhK*
	Cytochrome b/f complex	*petA*, *petB*, *petD*, *petG*, *petL*, *petN*
	ATP synthase	*atpA*, *atpB*, *atpE*, *atpF*, *atpH*, *atpI*
Other genes	Maturase	*matK*
	Subunit of acetyl-CoA carboxylase	*accD*
	Envelope membrane protein	*cemA*
	Protease	*clpP*
	C-type cytochrome synthesis	*ccsA*
	Conserved open reading frames	*ycf1*, *ycf2*
Peseudogene	Translation-related gene	*infA*

## ﻿Discussion

Similar to other *Hedyotis* species described previously (Wang et al. 2018; Jiang and Wang 2019; Xu and Wang 2021; Jiang and Wang 2021), the pollen grains of *H.longiramulis* are dimorphic between long-styled and short-styled flowers, i.e., the pollen of the short-styled flowers is larger than that of the long-styled flowers. This pattern was also found in other Rubiaceae with dimorphic flowers, e.g., *Damnacanthus* C.F.Gaertn. (Naiki and Nagamasu 2003) and *Arcytophyllum* Schult. & Schult.f. (Wolff and Liede-Schumann 2007).

The phylogenetic analysis shows that *H.longiramulis* is sister to *H.pubirachis* (Fig. 1), but it can be distinguished from this species by the puberulent stems and stipules (versus glabrous in *H.pubirachis*), the waxy leaf surface (versus non-waxy in *H.pubirachis*) and the inflorescences growing on long lateral branches (versus inflorescences on the main stem and lateral branches in *H.pubirachis*) (Table 3).

Comparing to chloroplast genome of the new species to that of *H.ovata*, we found that there was a 300 bp contraction that occurred in the IR regions of *H.longiramulis* excluding almost entirely the *rps19* gene from the IR/LSC boundaries (Fig. 5). However, we currently can’t predict the fluctuation tendency in this genus due to insufficient chloroplast genomic data. We suggest that the complete chloroplast genome would be informative and would help resolve infrageneric relationships within the genus.

### ﻿Key to the 24 *Hedyotis* species sampled in this study

**Table:** 

1	Stem terete or slightly flattened	**2**
–	Stem tetragonal or sulcate, or at least so when juvenile	**13**
2	Leaves ovate to ovate-triangular; inflorescences 1-flowered or 2–4-flowered and fasciculate	***H.pulcherrima***
–	Leaves lanceolate, ovate-lanceolate, or lanceolate-elliptic; inflorescences cymose or paniculate cymose	**3**
3	Stipules more or less puberulent abaxially	**4**
–	Stipules glabrous abaxially	**6**
4	Leaves densely puberulent on both sides	***H.puberulifolia***
–	Leaves glabrous on both sides or only puberulent on midrib adaxially	**5**
5	Position of inflorescences strictly axillary	***H.loganioides***
–	Position of inflorescences terminal and axillary in upper nodes	***H.tenuipes***
6	Position of inflorescences strictly axillary	***H.communis***
–	Position of inflorescences terminal and axillary in upper nodes	**7**
7	Inflorescences showing dichasial branching at sub-axes	**8**
–	Inflorescences showing monochasial branching at sub-axes	**10**
8	Inflorescence axes terete	***H.cantoniensis***
–	Inflorescence axes more or less 4-angled or sulcate	**9**
9	Peduncles hollow, slightly sulcate; corolla tubes ca. 2.5 mm long	***H.nankunshanensis***
–	Peduncles solid, 4-angled and sulcate; corolla tubes 3.0–4.0 mm long	***H.caudatifolia***
10	Inflorescence axes 4-angled and sulcate	***H.pubirachis***
–	Inflorescence axes terete	**11**
11	Leaves narrowly elliptic to lanceolate; stipules triangular	***H.nanlingensis***
–	Leaves ovate, broadly elliptic or lanceolate; stipules broadly triangular	**12**
12	Leaves ovate to lanceolate; capsules oblong-ellipsoid	***H.longiexserta***
–	Leaves ovate to broadly elliptic; capsules subglobose	***H.effusa***
13	Stems more or less puberulent or scabrous	**14**
–	Stems glabrous	**17**
14	Leaves base broadly rounded or amplexicaul; leaves densely pilose on both sides	***H.xanthochroa***
–	Leaves base cuneate, narrowly cuneate or shortly decurrent; leaves glabrous, waxy or puberulent on both sides	**15**
15	Leaf surface waxy on both sides; inflorescences growing at lateral branches	***H.longiramulis***
–	Leaves glabrous to puberulent on both sides; inflorescences growing at terminal and upper axillary of main stem	**16**
16	Flowers not enclosed by two ovate leaflike bracts; corolla white or purplish abaxially; corolla tubes shorter than 3 mm	***H.matthewii***
–	Flowers enclosed by two ovate leaflike bracts; corolla purple abaxially; corolla tubes longer than 15 mm	***H.yangchunensis***
17	Inflorescences axillary	**18**
–	Inflorescences terminal and axillary in upper nodes of stem	**19**
18	Stipules broadly triangular, margins not reflexed, apex apiculate to aristate	***H.interrupta***
–	Stipules ovate or triangular, margins becoming reflexed, apex acute to acuminate	***H.acutangula***
19	Corolla purple abaxially; corolla tubes longer than 5 mm	**20**
–	Corolla white or purplish abaxially; corolla tubes shorter than 3 mm	**21**
20	Leaves lanceolate, narrowly lanceolate or narrowly elliptic, scabrous	***H.exserta***
–	Leaves ovate, glabrous	***H.ovata***
21	Flowers homostylous; corolla tubes pilosulous adaxially	**22**
–	Flowers heterostylous; corolla tubes pubescent adaxially	**23**
22	Leaves narrowly lanceolate or lanceolate; corolla lobes longer than tube	***H.longipetala***
–	Leaves narrowly elliptic, elliptic or lanceolate; corolla lobes nearly equal to tube in length	***H.matthewii***
23	Stipules broadly triangular, glabrous abaxially	***H.consanguinea***
–	Stipules triangular, pubescent abaxially	**24**
24	Inflorescences at terminal and upper axillary of main stem; peduncles shorter than 5 cm	***H.xinyiensis***
–	Inflorescences at terminal of main stem; peduncles longer than 5 cm	***H.taishanensis***

## ﻿Conclusion

The new species of *Hedyotislongiramulis* is described based on the combination of morphological and molecular evidence. In addition, the micromorphological characters of seed, pollen and leaf epidermal features were illustrated.

## Supplementary Material

XML Treatment for Hedyotis longiramulis

## ﻿Additional information

### Author ORCIDs

Yi-Da Xu https://orcid.org/0000-0001-9812-4770

Ying Zhang https://orcid.org/0000-0002-8352-1543

Rui-Jiang Wang https://orcid.org/0000-0002-4985-8281

### Data availability

All of the data that support the findings of this study are available in the main text.
